# Driving lesson or driving test? A metaphor to help faculty separate feedback from assessment

**DOI:** 10.1007/s40037-020-00617-w

**Published:** 2020-09-09

**Authors:** Paul L. P. Brand, A. Debbie C. Jaarsma, Cees P. M. van der Vleuten

**Affiliations:** 1 Department of Medical Education and Faculty Development, Isala Hospital, Isala Academy, Zwolle, The Netherlands; 2 Lifelong Learning, Education and Assessment Research Network (LEARN), University Medical Centre Groningen, Groningen, The Netherlands; 3 Centre for Educational Development and Research (CEDAR), University Medical Centre Groningen, Groningen, The Netherlands; 4 Department of Educational Development and Research, Faculty of Health, Medicine and Life Sciences, Maastricht University, Maastricht, The Netherlands

**Keywords:** Feedback, Assessment, Programmatic assessment

## Abstract

Although there is consensus in the medical education world that feedback is an important and effective tool to support experiential workplace-based learning, learners tend to avoid the feedback associated with direct observation because they perceive it as a high-stakes evaluation with significant consequences for their future. The perceived dominance of the summative assessment paradigm throughout medical education reduces learners’ willingness to seek feedback, and encourages supervisors to mix up feedback with provision of ‘objective’ grades or pass/fail marks. This eye-opener article argues that the provision and reception of effective feedback by clinical supervisors and their learners is dependent on both parties’ awareness of the important distinction between feedback used in coaching towards growth and development (assessment for learning) and reaching a high-stakes judgement on the learner’s competence and fitness for practice (assessment of learning). Using driving lessons and the driving test as a metaphor for feedback and assessment helps supervisors and learners to understand this crucial difference and to act upon it. It is the supervisor’s responsibility to ensure that supervisor and learner achieve a clear mutual understanding of the purpose of each interaction (i.e. feedback or assessment). To allow supervisors to use the driving lesson—driving test metaphor for this purpose in their interactions with learners, it should be included in faculty development initiatives, along with a discussion of the key importance of separating feedback from assessment, to promote a feedback culture of growth and support programmatic assessment of competence.

## Feedback in clinical education: important, but still underused

Feedback is a key tool to support workplace-based learning in clinical medicine [1–3]. It helps learners at all stages of medical education to make the most of the experiential learning opportunities in encounters with patients [4, 5]. Clinical supervisors can use feedback to support learners’ growth towards increasing autonomy and independent practice by forming educational alliances with their learners [6], engaging with learners in informed self-assessment and reflection, and co-creating a safe learning environment with their learners [4, 7]. Recent research unravelling the complexities of feedback conversations in clinical education is thought to help clinical supervisors to provide frequent constructive feedback to their learners [2, 5, 7, 8], and to inform faculty development initiatives to improve clinical supervisors’ feedback conversation techniques [9–11].

Despite these advancements in our understanding of the usefulness and the optimal provision of feedback, medical learners continue to experience a limited amount of feedback during their clinical placements, receive feedback that is too general or limited in scope to be helpful, and engage with faculty deficient in feedback competencies [1, 5, 8, 12, 13]. Recent observations suggest that competency-based medical education creates tension between feedback intended to support a learner’s growth and the formal assessment procedures needed to assess the acquisition of the core competencies of the programme [13, 14]. Learners tend to perceive learning activities like direct observation of clinical skills as high-stakes evaluations with significant consequences for their future [14–17], prompting them to avoid feedback opportunities associated with direct observations [18–20], and hence missing out on the potentially very useful feedback associated with it. In addition, perceived time constraints prompt supervisors to avoid or opt out of directly observing their learners in performing relevant clinical skills [21], which further compromises the resident feedback-seeking behaviour [18–20].

At first sight, this appears to be a problem of learners’ behaviour. Thus, it would be tempting to try and tackle this problem by targeting the learners, by addressing and trying to modify their feedback-seeking behaviour [22]. In this eye-opener article, however, we argue that it is the clinical supervisors’ responsibility to ensure that supervisors and learners achieve a clear mutual understanding of the purpose of each of their interactions. Clinicians supervising medical learners in the clinical workplace must themselves be able to clearly distinguish feedback from assessment, to allow them to explain the difference to their learners, and to achieve the desired clear mutual understanding of the purpose of their encounter.

Although the importance of distinction between feedback and assessment has been stressed for more than 20 years [23], clinical supervisors continue to confuse and blend feedback and assessment to this day [5, 13, 24, 25]. This suggests that the methods to teach them about this distinction and its importance should be improved. In this paper, we present a metaphor which we have found very useful for this purpose in faculty development courses: the distinction between driving lessons and driving tests (Box 1).

### Box 1 The driving test metaphor—a personal account of the first author

On the day of my driving test, many years ago, I was feeling pretty nervous. After a series of lessons by a firm but friendly driving instructor, I was confident I could do all the manoeuvres required for the exam. My driving test, however, was scheduled during rush hour in a university city, with its masses of cyclists ignoring every traffic light and sign in sight, adding to the complexity of inner-city car and truck traffic. During the driving test, I had to brake suddenly on two occasions in response to other road users’ erratic behaviour. After the required 45 min of driving and parking/turning procedures, the examiner told me I had passed the test, which—obviously—made me happy and proud. He added, however, that it had been a close call. “Twice, I almost hit the emergency brake”, he said. “And you know that if I had had to do that, you would have failed the test”. Sure, I nodded, I know that. Then, he added, “If I were you, I’d take some more driving lessons to work on your approach to busy intersections. Strike a better balance between speed and safety in busy traffic”. I was confused for a moment. What did he mean to tell me here? Was my driving not good enough? If he really thought that, he should have failed me. But he didn’t. I double-checked to be sure and he confirmed that I had passed the test. So I thought, “If my driving today was good enough to pass the test, you can keep your advice and stick it, well, anywhere”. I collected my driving licence a few days later, started independent practice as a licensed driver, and got better and better over time (I think), with increasing practice and exposure.

Only many years later did I realise what had happened. The examiner had confused assessment and feedback. As a result, his feedback was ineffective.

## Responsibility of examiners in high-stakes assessments

The driving examiner’s task of assessing a candidate’s competence as a driver (Box 1) is an important responsibility: as a society, we trust that these examiners will make sure that incompetent drivers are not allowed on to our roads, for the benefit of other road users’ safety. We have a comparable responsibility to fail those medical students and residents who do not meet the minimal standards of competence that we have established for licensed medical doctors or specialists [26, 27].

Exams like a driving test or a licensing exam are high-stakes summative assessments of the learning that has taken place earlier. Like the secondary school and university education systems, the medical education culture is dominated by the primacy of the summative assessment paradigm [28, 29], which builds on the premise that (summative) assessment drives learning [25, 30]. Supervisors feel a strong responsibility to prevent unsafe learners qualifying as licensed doctors. Pass/fail tests and tests with grades are considered objective, rigorous and indispensable for learning by many supervisors and learners [25, 28], which helps in understanding their common use at all stages of medical education.

## What happens if supervisors and learners mix up assessment and feedback?

The first author’s experience with his driving test (Box 1), and his response to the examiner’s feedback, illustrates what happens if supervisors (and, as a consequence, their learners) mix up feedback and summative assessment. At a high-stakes exam like the driving test, most learners are not receptive to feedback [9, 31, 32]. They are in exam mode: all they want to do is pass the test and receive the positive feedback that they did a good job. This phenomenon has been described by various authors in medical education. Residents who perceive workplace-based assessments as high-stakes exams with potentially serious consequences for their future tend to ignore or discard the feedback associated with them [16, 33]. They “play the game” of seeking only positive feedback (i.e. only ask for feedback on a task or procedure they think they did well) [13, 31, 34]. They use these positive assessments to buff their portfolios. They believe that this proves their clinical competence, which will lead their supervisors to sign off on their entrustable professional activities, in-training evaluation reports, or annual progress assessments [13, 32]. Residents employ this and other impression management strategies to portray an image of competence [13, 25, 35]. They view direct observations of clinical skills as “staging a performance” in which they are expected to demonstrate a “textbook” example of competence [13, 15]. All these observations show that viewing a workplace-based assessment as a test, as a high-stakes exam, which many learners do, renders the learner unreceptive to feedback. In exam mode, we just want to perform, look good, and pass the test. Like the first author did at his driving test.

## What if we approached feedback in workplace-based learning like a driving lesson?

Conversely, people tend to be very receptive to feedback during driving lessons. These are clearly identified as low-stakes learning opportunities. Most driving instructors are patient in coaching candidates towards mastering the complex skills and procedures of driving a car in everyday traffic. Like in other coaching relationships, failures during driving lessons are embraced as catalysts for learning [36]. During driving lessons, most candidates are eager to hear their driving instructor’s feedback, because it helps them to improve their driving skills. They are in learning mode.

Being in learning mode helps people to use the feedback given to improve their performance, develop and grow [37]. Feedback framed as repeated coaching over time aimed at improving clinical skills promotes the acceptance of feedback and acting upon it [36, 38]. Designing feedback as a dialogue gives learners the opportunity to take ownership of their strengths and weaknesses [8]. Particularly when the feedback comments given are specific, detailed, take into consideration what effect the feedback will have, and are personalised to the learner’s own work, this will help learners to change their behaviour and improve their performance [9, 39].

## Usefulness of the metaphor

The beauty of the driving test—driving lesson metaphor lies in its degree of recognition. Everybody knows the stress and anxiety involved in high-stakes exams like the driving test. Even the rare adult without a driving licence has friends or relatives who have experienced it. Everyone understands the difference between the learning in driving lessons and the performance during the driving test. In our experience in faculty development sessions, the driving test—driving lesson metaphor helps clinical teachers to appreciate the learners’ difficulty in accepting feedback when they are in exam mode, and the learners’ receptivity to feedback when they are in learning mode. The simple metaphor illustrates the key difference between exam and learning mode (or between performance and learning goal orientation) without having to resort to complex educational jargon that may confuse and irritate physicians [40].

The metaphor also helps in understanding the value of a long-term coaching relationship between learner and supervisor. If a supervisor (like a driving instructor) succeeds in supporting the learner to trust him (or her), this will increase the learner’s willingness to accept the feedback and learn [9, 13, 37, 41].

Finally, the metaphor helps in appreciating that, like the clinical supervisor, the driving instructor’s main task is to provide feedback aimed at promoting the learner’s driving, not passing judgement on its quality.

## The dual role of programmatic assessment

Appreciating that there is no single reliable test to assess competence in workplace-based learning, the term “programmatic assessment” was coined to describe a deliberate programme of different assessment methods, which alleviates the limitations of each individual assessment [42]. Although this model has received widespread support in educational research [29], its implementation in competency-based medical education practice remains challenging [13, 14, 30]. A key difficulty remains the dual role of programmatic assessment, serving both learning and decision-making functions [29]. Whilst assessment in each individual encounter between a learner and a supervisor is used as a basis to provide feedback to foster the learner’s growth and development (assessment for learning, low stakes), a final assessment with a pass/fail decision is made after a coherent interpretation across many assessment methods (assessment of learning, high stakes) [29]. Most clinical supervisors realise that such an overall judgement of competence requires information from multiple sources and supervisors [43]. They understand that each supervision encounter with a student or resident is just a snapshot impression, which does not necessarily reflect the learner’s overall competence [29]. However, the pervading primacy of the summative assessment paradigm throughout the medical education continuum makes it difficult to remove formal assessment contamination from feedback aimed at promoting the learner’s growth [13]. The driving test—driving lesson metaphor can help to make the distinction between these two functions of programmatic assessment, and support the desired increase of the formative function of each individual workplace-based assessment.

Even in a programmatic assessment programme designed to support the development of competence by coaching in the moment and coaching over time, in which each encounter between learner and faculty is set up to support learning [44], all information collected by supervisors during these encounters will be used to create the most accurate representation of the learner’s competence development over time [29, 42]. Realising this contamination of purpose should not discourage faculty from pursuing maximum separation of feedback and assessment.

The driving lesson—driving test metaphor makes the dual function of programmatic assessment more understandable to clinical supervisors. Although each driving lesson is used to coach candidates towards increasing driving competence, the driving instructor decides the moment at which the candidate is ready to enrol for the driving test. At that point in time, the driving instructor expresses the confidence that the candidate is a sufficiently competent driver. He (or she) has seen the candidate in driving action on so many occasions that he (or she) feels confident to reliably assess the candidate’s competence. Similarly, in medical education, clinical supervisors can use each feedback and coaching session as an individual data point, and use all these data points together to paint an increasingly clear picture of the student’s or resident’s emerging competence as a doctor in the field of training [29].

## Limitations of the metaphor

The limitations of the driving lesson—driving test metaphor need to be taken into account. First, countries and programmes differ in their approach to high-stakes summative assessment of competence as a doctor or medical specialist. Some apply formal licensing exams at the end of a programme or curriculum, assessing both knowledge and clinical skills, which are easily comparable to driving theory and practice tests. Others, like the Netherlands, use the overall judgement of the programme director or supervisory team as the final high-stakes summative assessment of competence. Although this is less directly comparable to a driving test, it is our experience in faculty development sessions that the driving test—driving lesson metaphor helps supervisors and residents to realise that they tend to mix up assessment and feedback, and why this is undesirable. Second, in many competency-based medical education curricula, the same supervisors play a role both in assessment for learning (feedback, low stakes) and assessment of learning (high stakes), whilst the roles of driving instructor and examiner are strictly separated in most countries. In addition, all driving lessons are almost always given by the same driving instructor, as compared to the large number of clinical supervisors involved in the coaching of medical students and residents in most clinical teaching departments today [45]. This makes it even more important for there to be a relationship of mutual trust between resident and supervisors [9, 41], and that the overall judgement of clinical competence is made by the entire group of supervisors [43, 46]. Thirdly, a system of programmatic assessment, in which formative feedback and summative assessment encounters are clearly separated, should be supported by system factors applying the same distinction. These include a clear institutional or departmental vision on the goals and structure of the programmatic assessment programme [11, 44], the use of forms avoiding contamination of feedback and assessment (e.g. a feedback form that does not require the supervisor to provide an overall grade or global rating of competence) [47], and a portfolio which clearly separates those components which are being used for high-stakes assessments (e.g. having fulfilled a minimum number of procedures and the results of mandatory knowledge tests) from those intended to support the learner’s growth as a doctor in training [48, 49]. This can be challenging, when department or institution leaders themselves struggle with the distinction between feedback and assessment, or when the forms and portfolios used are chosen by convenience and tradition instead of being purposely designed. We encourage supervisors to be civilly disobedient when they are prompted to complete a form which mixes up feedback and assessment, and only to use the part of the form that fits the mutually agreed purpose of the encounter with the learner. Finally, although our experience in using this metaphor in faculty development courses is consistently positive, empirical studies are needed to test the hypothesis that using this metaphor in faculty development affects participating faculty’s behaviour in their practice of providing feedback.

## Using the metaphor to promote programmatic assessment in faculty development

Numerous authors have argued and shown that faculty development training is needed to promote effective feedback in meaningful conversations between supervisors and learners [9–11, 44, 50–52]. The implications of the importance of distinguishing between feedback to support learning (low stakes) and judgement (high stakes) for faculty development courses are presented in Tab. 1.

**Table 1:** Implications for faculty development initiatives of distinguishing feedback from assessment

Principle	Use in faculty development
Clearly distinguish between assessment for learning (feedback) and assessment of learning (high-stakes test)	Use a metaphor, like the difference between driving lessons and the driving test, to enable faculty to appreciate the difference between the two
Feedback focused on improvement as the guiding principle of clinical supervision	Build on the driving lesson metaphor: each encounter between learner and supervisor can be viewed (and framed) as a driving lesson, with the supervisor in the role of the driving instructor (not examiner)
Identification of underperformance (i.e. insufficient competence) requires input from multiple sources and, hence, a group judgement	Discuss how the group of supervisors can design methods to collect and collate data from multiple supervisors and encounters to form a clear picture of the learner’s (growth in) competence
Importance of department feedback culture on feedback delivery routines and learners’ receptivity to feedback	Support the supervisors of a clinical teaching department to develop a department feedback culture aimed at promoting growth and development
Teach feedback delivery techniques that support the principles of effective coaching to all supervisors	Emphasise that effective feedback is a conversation built on trust and mutual engagement, not a one-way delivery of information
Use forms and portfolios that support the distinction between feedback and assessment	Avoid forms which contain both narrative feedback elements and overall assessments of competence or grades
Clear objectives and expectations regarding the principles of feedback	Teach programme directors to discuss the distinction between feedback and assessment with the supervisors and the learners in the department. Ensure that all supervisors understand this principle and encourage them to act accordingly

The arguments laid out in this paper support the notion that such faculty development initiatives should include a discussion of the importance of separating feedback from formal high-stakes assessment [27], and promote coaching, rather than judgement, as the guiding principle of clinical supervision [36, 44]. It is the programme director’s responsibility to ensure that the entire faculty shares the view that feedback is a tool to support learners’ growth and development, and to promote a feedback culture of growth [11, 37]. We recommend that programme directors discuss the principles of direct observation and coaching with each learner entering their department, and address this in meetings of the team of supervisors, to ensure that each of them understands the difference between feedback and assessment, and knows that their role is to provide low-stakes feedback aimed at promoting the learner’s growth, and not high-stakes judgement, in each encounter with a learner [24]. Using the driving lesson—driving test metaphor helps to highlight that each interaction between the learner and a supervisor is to be seen as a driving lesson, as an opportunity to learn, and not as a high-stakes exam. It is our experience that this distinction contributes to a safe learning environment in which leaners are increasingly willing to show their vulnerability and acknowledge points for improvement [41]. Learners should be made aware that the decision on pass/fail assessments will be made within the group of supervisors, and is not based solely on the assessment forms recorded in the portfolio [13, 32]. Forms capturing feedback and stored in portfolios should be purposely designed to reflect their feedback purpose, and be devoid of summative grades and overall competence assessments. Learners should also be reassured that such high-stakes judgements can never come as a surprise, because any concern among the team of supervisors about the learner’s performance or growth in competence will be discussed with the learner at an early stage [9, 28]. All supervisors should be trained in methods for effective feedback conversations, highlighting the importance of trust and mutual engagement in these conversations [7, 8, 53, 54].

## Conclusions

To promote the provision of effective feedback by clinical supervisors and the receptivity of medical learners to feedback, both feedback providers and recipients should be aware of the important distinction between coaching towards growth and development (feedback, assessment for learning) and reaching a judgement on the learner’s competence and fitness for practice (high-stakes exam, assessment of learning). Using driving lessons and the driving test as a metaphor for feedback and assessment may help supervisors and learners to understand this crucial difference and to act upon it. This metaphor can be used in faculty development initiatives to promote a feedback culture of growth, and to support programmatic assessment of competence.
